# Proteomic Signature of Host Response to SARS-CoV-2 Infection in the Nasopharynx

**DOI:** 10.1016/j.mcpro.2021.100134

**Published:** 2021-08-14

**Authors:** Patrick M. Vanderboom, Dong-Gi Mun, Anil K. Madugundu, Kiran K. Mangalaparthi, Mayank Saraswat, Kishore Garapati, Rana Chakraborty, Hideki Ebihara, Jie Sun, Akhilesh Pandey

**Affiliations:** 1 Department of Laboratory Medicine and Pathology, Division of Clinical Biochemistry and Immunology, Mayo Clinic, Rochester, Minnesota, USA; 2 Institute of Bioinformatics, International Technology Park, Bangalore, Karnataka, India; 3 Manipal Academy of Higher Education, Manipal, Karnataka, India; 4 Center for Molecular Medicine, National Institute of Mental Health and Neurosciences, Bangalore, Karnataka, India; 5 Amrita School of Biotechnology, Amrita Vishwa Vidyapeetham, Kollam, Kerala, India; 6 Division of Pediatric Infectious Diseases, Mayo Clinic, Rochester, Minnesota, USA; 7 Department of Obstetrics and Gynecology, Mayo Clinic, Rochester, Minnesota, USA; 8 Department of Molecular Medicine, Mayo Clinic, Rochester, Minnesota, USA; 9 The Robert and Arlene Kogod Center on Aging, Mayo Clinic, Rochester, Minnesota, USA; 10 Department of Immunology, Mayo Clinic, Rochester, Minnesota, USA; 11 Division of Pulmonary and Critical Medicine, Department of Medicine, Mayo Clinic, Rochester, Minnesota, USA; 12 Center for Individualized Medicine, Mayo Clinic, Rochester, Minnesota, USA

**Keywords:** phosphoproteomics, virus, infection, upper airway, COVID-19, interferon, host response, AGC, automatic gain control, COVID-19, coronavirus disease 2019, FDR, false discovery rate, IFN, interferon, ISGs, interferon-stimulated genes, MERS-CoV, Middle East respiratory syndrome coronavirus, MPB, mobile phase B, NHBE, normal human bronchial epithelial, NP, nasopharyngeal, OGFR, opioid growth factor receptor, OPRPN, Opiorphin prepropeptide, PPIase, peptidyl-prolyl *cis*/*trans* isomerase, PRM, parallel reaction monitoring, PRRs, pathogen-recognition receptors, SARS-Cov-2, severe acute respiratory syndrome coronavirus 2

## Abstract

Coronavirus disease 2019 (COVID-19), caused by severe acute respiratory syndrome coronavirus 2 (SARS-CoV-2) infection, has become a global health pandemic. COVID-19 severity ranges from an asymptomatic infection to a severe multiorgan disease. Although the inflammatory response has been implicated in the pathogenesis of COVID-19, the exact nature of dysregulation in signaling pathways has not yet been elucidated, underscoring the need for further molecular characterization of SARS-CoV-2 infection in humans. Here, we characterize the host response directly at the point of viral entry through analysis of nasopharyngeal swabs. Multiplexed high-resolution MS-based proteomic analysis of confirmed COVID-19 cases and negative controls identified 7582 proteins and revealed significant upregulation of interferon-mediated antiviral signaling in addition to multiple other proteins that are not encoded by interferon-stimulated genes or well characterized during viral infections. Downregulation of several proteasomal subunits, E3 ubiquitin ligases, and components of protein synthesis machinery was significant upon SARS-CoV-2 infection. Targeted proteomics to measure abundance levels of MX1, ISG15, STAT1, RIG-I, and CXCL10 detected proteomic signatures of interferon-mediated antiviral signaling that differentiated COVID-19-positive from COVID-19-negative cases. Phosphoproteomic analysis revealed increased phosphorylation of several proteins with known antiviral properties as well as several proteins involved in ciliary function (CEP131 and CFAP57) that have not previously been implicated in the context of coronavirus infections. In addition, decreased phosphorylation levels of AKT and PKC, which have been shown to play varying roles in different viral infections, were observed in infected individuals relative to controls. These data provide novel insights that add depth to our understanding of SARS-CoV-2 infection in the upper airway and establish a proteomic signature for this viral infection.

## Highlights

• Nasopharyngeal swabs are an excellent sample to study host response in COVID-19.
• Proteins encoded by interferon-stimulated genes were robustly upregulated.
• Novel host response proteins were revealed by proteomics and phosphoproteomics.
• Host phosphoproteome is substantially altered upon SARS-CoV-2 infection.

## Graphical Abstract

**Figure:**
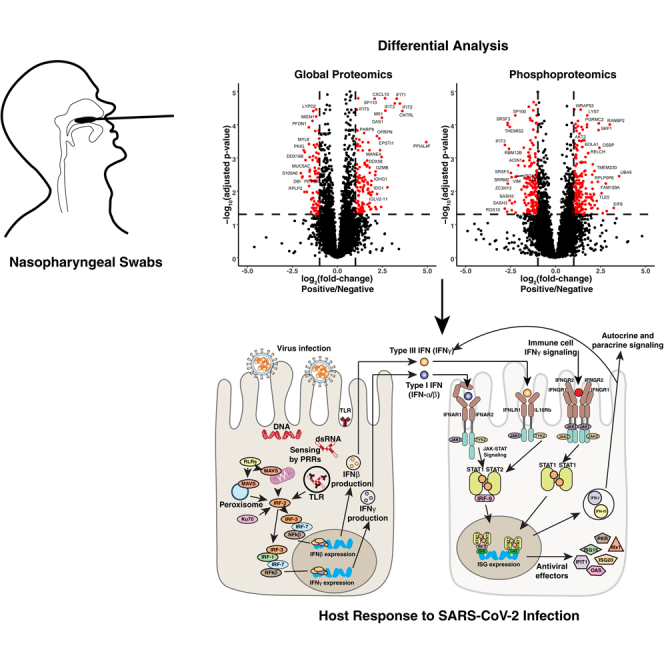

## In Brief

Dysregulated immune response to SARS-CoV-2 infection underlies severe diseases; however, the precise underlying mechanisms have not yet been elucidated. To establish a proteomic signature of SARS-CoV-2 infection in the upper airway, the authors of this study performed a deep proteomic and phosphoproteomic analysis of nasopharyngeal swabs. This study is a valuable resource for investigators interested in studying host response to SARS-CoV-2 infection.

Coronaviruses are enveloped, nonsegmented, positive-sense RNA viruses of the family Coronaviridae and subfamily Coronavirinae. Human alpha-coronavirus infections have traditionally been associated with mild upper respiratory symptoms and account for a large percentage of “common colds” (1, 2, 3). Over the past 18 years, however, three separate beta-coronaviruses have crossed the species barrier to become lethal zoonotic human pathogens. Severe acute respiratory syndrome coronavirus 2 (SARS-Cov-2), which emerged in 2002, infected 8098 individuals resulting in 744 deaths. In 2014, the Middle East respiratory syndrome coronavirus (MERS-CoV) infected 2494 individuals resulting in 898 fatalities (4). SARS-CoV-2, the virus responsible for coronavirus disease 2019 (COVID-19), has already resulted in a global pandemic with millions of people infected worldwide.

**Fig. 1:**
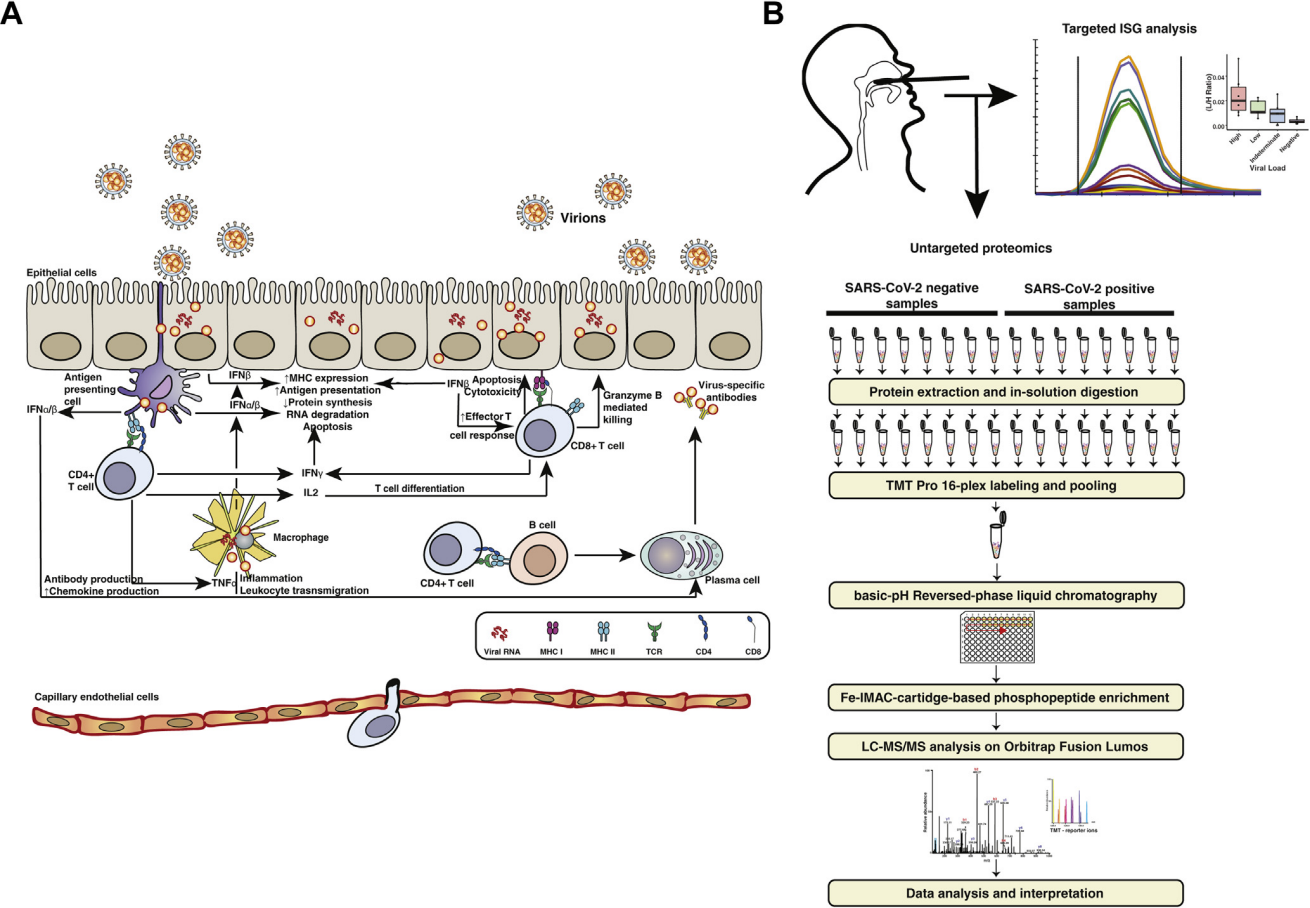
**Experimental strategy.***A*, schematic of innate and adaptive immune response to viral infection. Infected nonimmune cells recognize viral pathogen-associated molecular patterns (PAMPs) *via* RIG-like receptors (RLRs) and toll-like receptors (TLRs) and produce IFNβ, while antigen presenting cells (APCs) such as plasmacytoid dendritic cells (pDCs) process infected cell debris and produce IFNα. By autocrine and paracrine action, these type I IFNs lead to the expression of interferon-stimulated genes (ISGs), which lead to an intracellular antiviral response. They also lead to increased capillary permeability, leukocyte recruitment, Th0 cell differentiation, and NK cell mediated killing of infected cells. T cell receptor (TCR)-mediated antigen detection by CD4+ T cells leads to the release of proinflammatory cytokines including type II IFN (IFN-γ) and effector T cell recruitment. *B*, schematic of experimental workflow. For the discovery workflow, protein was extracted from nasopharyngeal swabs (RT-PCR positive: n = 8, RT-PCR negative: n = 8) digested with trypsin and fractionated using high-pH reversed-phase fractionation. Peptides without any enrichment were subjected to global proteome analysis, and peptides subjected to phosphopeptide enrichment using immobilized metal-affinity chromatography (IMAC) were analyzed separately as shown. Targeted PRM analysis of separate NP swab samples (high viral load: n = 6, low viral load: n = 6, negative: n = 6, indeterminate: n = 6) was carried out to monitor levels of several key interferon-stimulated genes (ISGs). IFN, interferon; NP, nasopharyngeal.

Interferon (IFN)-mediated antiviral response forms the first line of defense against viral pathogens in which the infected host epithelial cells recognize viral pathogen-associated molecular patterns such as uncapped mRNA or replication intermediates (5) *via* cytosolic (RIG-I, MDA5) or endosomal (TLR3, TLR4) pathogen-recognition receptors (PRRs) (Fig. 1*A*). Virus recognition by PRRs triggers a cascade of downstream signaling events leading to activation of transcription factors nuclear factor kB (NF-kB) and IFN regulatory factors 3 and 7 (IRF3 and IRF7), mediating the expression of proinflammatory cytokines (e.g., IL-1, IL-6, TNF-α), type I (IFN-α, IFN-β) and type III IFNs (IFN-λ), respectively (6). Infected cells secrete IFNs, which signal in an autocrine and paracrine manner, to establish an antiviral state in the cells through inhibition of viral transcription, translation, and replication, as well as degradation of viral nucleic acids and recruitment of immune cells (7). Although the transcriptional responses to type I and type III IFNs are similar, cell type–specific expression of receptors and signaling kinetics leads to distinct responses. Type III IFN (IFN-λ) signaling is less potent, slowly induced but more sustained relative to the type I IFN response, which is more potent, rapidly induced, and transient (6, 8). Most nucleated cells express heteromeric cell surface receptors (IFNAR1 and IFNAR2) that mediate responses to type I IFNs. Epithelial cells, however, preferentially express the heteromeric receptors for IFN-λ (IFNLR1 and IL10Rβ). Thus, type III IFN signaling plays an important role in antiviral protection of the upper and lower respiratory, gastrointestinal, and reproductive tracts (8, 9, 10, 11).

SARS-CoV and MERS-CoV exploit various strategies to mitigate the host immune response. SARS-CoV-encoded nsp1 protein inhibits host gene expression by promoting host mRNA degradation (12), while papain-like protease and ORF3b proteins inhibit IRF3 phosphorylation and prevent its translocation to the nucleus. Papain-like protease also inhibits NFκB activation by preventing the degradation of IκBα (13, 14). Furthermore, MERS-CoV ORF 4a, 4b, and 5 proteins antagonize IFN production by inhibiting nuclear localization of IRF3, thereby preventing IFN promotor activation (15). SARS-CoV-2 is genetically more similar to SARS-CoV, sharing 79% of its genomic sequence identity compared with MERS, which shares only 50% genomic sequence identity to SARS-CoV-2 (16). Despite genomic differences among the three human beta-CoVs, their clinical features are similar. Common symptoms of COVID-19 include fever, dry cough, and fatigue; however, severe cases can result in pneumonia, acute respiratory distress syndrome, multiorgan failure, and death (4, 17). Similar to SARS and MERS, increasing age and underlying health comorbidities including diabetes, obesity, and heart failure are associated with increased risk of poor outcomes in patients infected with SARS-CoV-2 (17, 18, 19, 20, 21, 22, 23, 24, 25).

Previous studies of SARS and MERS indicate that direct cytopathic effects caused by these viruses along with evasion of host immune response are factors affecting disease severity (26, 27). A number of recent reports have sought to characterize SARS-CoV-2 infection in an effort to understand its pathogenesis, identify prognostic markers, and discover potential therapies. Accurate and specific methods of SARS-CoV-2 detection by MS have been developed (28, 29, 30, 31, 32) to complement RT-PCR–based testing. Interaction of host proteins with virus-encoded proteins have been elucidated using cell culture experiments, and potential drugs to target these interactions have been identified (33). Multi-omics analysis of peripheral blood samples from COVID-19 patients utilizing RNA-Seq and high-resolution MS identified over 200 molecular features with high correlation to COVID-19 status and severity (34). Changes in protein phosphorylation upon SARS-CoV-2 infection in an African green monkey kidney cell model (Vero E6 cells) suggested that a major portion of the host response to SARS-CoV-2 infection is driven by phosphorylation signaling (35). In addition, peripheral blood mononuclear cells from patients hospitalized for COVID-19 display heterogeneous interferon-stimulated gene (ISG) signatures, downregulation of HLA class II molecules, and a neutrophil population that may be characteristic of acute respiratory distress syndrome (36).

Both clinical and animal model studies have shown that a dysregulated immune response resulted in hyperinflammation–induced organ damage, a major contributing factor to disease severity in SARS and MERS (18, 37). Transcriptional profiling of cell and animal model systems has shown that SARS-CoV-2 pathology is driven by an elevated inflammatory signature characterized by low levels of type I and type III IFNs combined with increased levels of chemokines (38). Consistent with these results, a transcript level comparison of white blood cells from infected subjects with varying disease severity found that severe and critical cases are associated with impaired type1 IFN signaling and an increased inflammatory response (39). Quantitative proteomics analysis of urine from SARS-CoV-2–positive individuals also showed dysregulated immune response with decrease in abundance of several proteins indicative of renal damage such as NPHS2, SLC36A2, and SLC5A10 (32). In addition, a single-cell RNA sequencing study using nasopharyngeal (NP) swabs demonstrated that COVID-19 severity correlates with increased epithelium–immune cell interactions (40).

Because the exact mechanisms underlying this dysregulated immune response have not yet been determined, we sought to characterize the proteomic and phosphoproteomic response to SARS-CoV-2 infection directly at the point of infection using clinical samples with multiplexed untargeted proteomics. Our data demonstrate a robust IFN-mediated antiviral response in the nasopharynx of SARS-CoV-2-positive individuals and additionally identified several upregulated proteins not previously associated with antiviral responses. Phosphoproteomic analysis revealed regulation of several molecules with known antiviral effects and downregulation of several important signaling molecules including AKT and PKC. These data also implicate opioid growth factor receptor (OGFR) signaling in SARS-CoV-2 infection for the first time. Finally, through targeted mass spectrometric analysis of several key molecules involved in the IFN-mediated response, we demonstrate that levels of these antiviral and proinflammatory responses can help monitor the infection and may be of prognostic value.

## Experimental Procedures

### Experimental Design and Statistical Rationale

For the global and phosphoproteomics studies, protein was extracted from a total of 16 biological replicate NP swabs, eight of which were from individuals with confirmed SARS-CoV-2 by RT-PCR and eight of which were from negative controls. Subject information for SARS-CoV-2-infected individuals is summarized in supplemental Table S1. Samples were labeled with TMT-Pro and analyzed in the MaxQuant software environment, and differential analysis was carried out using a generalized linear model as described previously (41). For the targeted analysis, NP swabs from 24 additional biological replicates were analyzed (negative controls, n = 6; low viral load, n = 6, high viral load, n = 6; indeterminate, n = 6). All targeted data were analyzed in Skyline (42), and Student's *t* test was used to test for differences between groups using stable isotope–labeled internal standard normalized peak areas.

### NP Swab Specimen Collection and Handling

COVID-19-positive and COVID-19-negative NP swabs were selected from samples submitted to the Clinical Microbiology Laboratory at the Mayo Clinic in Rochester, Minnesota, for routine testing. All samples were collected in PBS and obtained with approval from the Mayo Clinic Institutional Review Board. The work described in this study abides by the Declaration of Helsinki principles.

### Protein Extraction and Digestion

NP swab samples were thawed and immediately treated with HALT phosphatase inhibitor cocktail (Thermo Scientific). As the NP swabs used for this experiment were residual clinical samples, the volume remaining for each was variable and ranged between 1 and 2 ml of PBS each. Each sample was aliquoted into multiple microcentrifuge tubes (200 μl aliquots), and protein was precipitated with the addition of methanol to a final concentration of 90%. Samples were then centrifuged at 15,000*g* for 10 min, and protein pellets from each sample were resuspended in a total of 200 μl of 8 M urea in 100 mM Tris, pH 8.0. Samples were then probe-sonicated to ensure each pellet was completely dissolved, and the protein content was estimated by BCA assay (Pierce). Disulfide bonds were reduced and alkylated with 10 mM TCEP and 10 mM IAA at room temperature for 30 min while protected from light. Trypsin/Lys-C Mix (Promega) was added at a 1:50 enzyme-to-protein ratio, and samples were digested overnight (16 h) at 37 °C. The enzymatic digest was terminated by adding TFA to a final concentration of 0.2%, and peptides were desalted using a 10 mg Strata-X PSVDB cartridge (Phenomenex), dried, and reconstituted in 100 μl of 50 mM TEAB. Peptide concentration was determined using a Pierce Colorimetric peptide assay, and 95 μg from each sample was labeled with TMTpro (Thermo Scientific) for 1 h.

### High-pH Reversed-Phase Fractionation

TMT-labeled peptides were fractionated by high-pH reversed-phase liquid chromatography on Dionex Ultimate 3000 (Thermo Scientific). Peptides (1.6 mg) were separated on a 4.6 mm × 50 cm × 3.5 μm Xbridge column (Waters) with a 2-h gradient from 2 to 40% mobile phase B (MPB). Mobile phase A was composed of 20 mM ammonium formate in water, and MPB was composed of 20 mM ammonium formate in 80% acetonitrile. A total of 96 fractions were collected and concatenated into 24 fractions. A 20-μg equivalent of each fraction was set aside for global proteome analysis, and the rest of each sample was concentrated into 12 fractions and dried before phosphopeptide enrichment.

### Phosphopeptide Enrichment

Each fraction was reconstituted in 100 μl of 80% acetonitrile in 0.1% TFA and transferred to a 96-well plate. IMAC-based phosphopeptide enrichment was performed using Fe(III)-NTA resin (G5496-60082) on an AssayMAP Bravo (Agilent Technologies) platform following the manufacturer's instructions.

### LC-MS/MS Analysis

Fractionated, TMT-labeled peptides (5 μg) were loaded onto a 20 mm × 0.075 mm PepMap C_18_ trap column with loading solvent composed of 0.1% TFA flowing at 20 μl/min using an Ultimate 3000 nano HPLC (Thermo Scientific). After 4 min, the ten-port valve was switched and peptides were eluted off the trap column onto a 50 cm × 75 μm ID PepMap EASY-Spray analytical column and peptides were separated with a 2 h gradient from 3 to 40% MPB. At 2 h, MPB was increased to 80% over 10 min, held for 15 min, and then returned to 3% for 5 min to re-equilibrate the column. The flow rate was set to 300 nl/min. Eluting peptides were analyzed on an Orbitrap Fusion Lumos (Thermo Scientific) mass spectrometer operated in a data-dependent mode. MS1 scans were acquired from 370 to 1700 *m/z* with an orbitrap resolution of 60,000 (at 200 *m/z*) at least every 3 seconds or when no more peptides were scheduled for fragmentation. The automatic gain control (AGC) was set at 1e6, and maximum ion fill time was set to 50 ms. The most abundant peptides with a charge state between 2 and 7 were selected for fragmentation with high-energy collision-induced dissociation using a normalized collision energy of 35% and a quadrupole isolation width of 0.7 *m/z*. The orbitrap resolution was set to 30,000, the maximum injection time was set to 54 ms, and the AGC target was set to 2e5, whereas the dynamic exclusion was set to 30 s using a 10 ppm mass window. For phosphopeptide analysis, the resolution for MS/MS was set to 50,000, and the maximum injection time was set to 86 ms.

### Data Analysis

MS raw files were processed in MaxQuant software, version 1.6.7.0 (43). Peptides were searched using the Andromeda search engine against the human UniProt FASTA database downloaded on July 24, 2019, concatenated with SARS-CoV-2, SARS-CoV, and MERS proteins for a total of 20,499 protein sequences. Enzyme specificity was set to trypsin/P, and maximum missed cleavages was set to 2 or less. Mass tolerance for the first search was set to 20 ppm and 4.5 ppm for the main search. Cysteine carbamidomethylation was set as a fixed modification, and protein N-terminal acetylation, methionine oxidation, and phosphorylated serine, threonine, and tyrosine were set as variable modifications. Searches were performed with a false discovery rate (FDR) of 1% for both peptides and proteins using a target–decoy approach. The peptide length was to at least seven amino acids long, and MS2 match tolerance was set to 0.02 Da. Enzyme specificity was set to trypsin, and a maximum of 2 missed cleavages were allowed. Protein data were extracted from the “proteinGroups.txt” file, and differential quantitation was carried using a generalized linear model as previously described (41).

### Targeted PRM Analysis

For targeted parallel reaction monitoring (PRM) analysis, samples were prepared as stated above for global and phosphoproteomic analysis except phosphatase inhibitors were not used. Stable isotope–labeled internal standards with isotopically labeled C-terminal arginine or lysine were synthesized by the Mayo Clinic Proteomics Core and spiked into each sample. Peptides were separated using a nano-HPLC set up identical to the configuration described above. For all samples, 250 ng of protein was loaded on to the trap column with the loading solvent flowing at 20 μl/min and washed for 4 min. The valve was then switched, and the peptides were eluted onto the analytical column and separated with a 20-min gradient from 2 to 40% MPB. Target peptides were monitored using an Orbitrap Eclipse (Thermo Scientific) mass spectrometer using an inclusion list with scheduled retention times. The parameters for the PRM scan were set as follows: AGC target was set to 5e4, maximum injection time was set to 54 ms, orbitrap resolution was set to 30,000, high-energy collision-induced dissociation normalized collision energy was set to 28%, and the quadrupole isolation was set to 1.6 m/z. The chromatographic peaks were manually integrated using the Skyline-daily software package. Peak areas for the top three to five most intense fragment ions from each target peptide (Table 1) were exported and summed for relative quantitation between groups using a Student's *t* test in R, version 3.6.3.

**Table 1:** List of peptides used for targeted PRM of ISGs.

Protein	Peptide	Light precursor (m/z)	Precursor charge	Heavy precursor (m/z)	Fragments monitored	Used for quantitation
CXCL10	VEIIATMK	452.7622	2	456.7693	y4, y5, y6	x
MX1	DVPDLTLIDLPGITR	819.4591	2	824.4632	y7, y8, y9, y10	x
	IFFENHPYFR	457.2278	3	460.5639	y3, y4, y5, y6	
ISG15	IGVHAFQQR	528.2909	2	533.295	y4, y5, y6, y8	x
	LTQTVAHLK	505.8033	2	509.8104	y5, y6, y7, y8	
STAT1	VMAAENIPENPLK	853.9649	2	717.3811	y7, y8, y9, y10, y11	x
	LLGPNASPDGLIPWTR	713.374	2	858.969	y7, y9, y10, y11	
DDX58	VVFFANQIPVYEQQK	905.4803	2	909.4874	y7, y8, y10, y11	x
	TNQNTGMTLPAQK	702.351	2	706.3581	y4, y8, y9, y10	

Targeted proteins and corresponding peptides are listed in each row. Parameters such as precursor ion m/z, charge, and fragment ions monitored are reported for each peptide. Stable isotope–labeled amino acids for each internal standard peptide are shown colored in red. Peptides from each protein that were used for relative quantitation between groups are indicated.

### Gene Set Enrichment Analysis

Gene set enrichment analysis was performed using Broad Institute's GSEA software. All gene sets that were negatively enriched in the COVID-19-positive subjects with an FDR-corrected *p*-value (using the Benjamini–Hochberg procedure) < 0.05 were reported.

### Motif Analysis

The peptide sequences surrounding the phosphorylation site of significantly downregulated entities (7 amino acids on each side of the phosphorylation site) were extracted from the ‘Phospho (STY)Sites.txt’ MaxQuant output file. Enriched motifs were identified using the Meme Suite online resource (http://meme-suite.org/) with the motif-x algorithm. The significance threshold was set to *p* < 1e-4, and the minimum occurrence of each motif was set to 20. The high-throughput NetworKIN (https://networkin.info/index_ht.shtml) tool was used to identify predicted protein kinases for all phosphosites with a fold change > ±1 and an adjusted *p*-value of <0.05. Kinases predicted to phosphorylate the enriched motifs identified using the motif-x algorithm were extracted from the NetworKIN output.

## Results and Discussion

### NP Swabs for Studying Host Immune and Epithelial Cells at the Point of SARS-CoV-2 Infection

Upon viral infection, interactions between immune and epithelial cells result in transcriptional activity that is cell type specific and integral to host–pathogen defense (44, 45). Dysregulation of these interactions can be deleterious, resulting in severe cases of COVID-19 (40). NP swabs are comprised of a heterogeneous mixture of cells of both epithelial and immune origins (40, 46). The nasopharynx is also the point of infection for SARS-CoV-2 and in close proximity to the lungs, where the majority of tissue damage occurs in severe cases of COVID-19. Therefore, NP swabs are an excellent and also accessible sample to study SARS-CoV-2 infection. We hypothesized that a comparative analysis between SARS-CoV-2-positive samples and negative controls should shed light on unique molecular features involved in the host response to SARS-CoV-2 infection. To this end, NP swab samples from patients who underwent PCR-based molecular testing were chosen for analyzing host responses to SARS-CoV-2. This included eight individuals who tested positive and eight individuals who tested negative.

### Quantitative Proteomic Analysis by LC-MS/MS

**Fig. 2:**
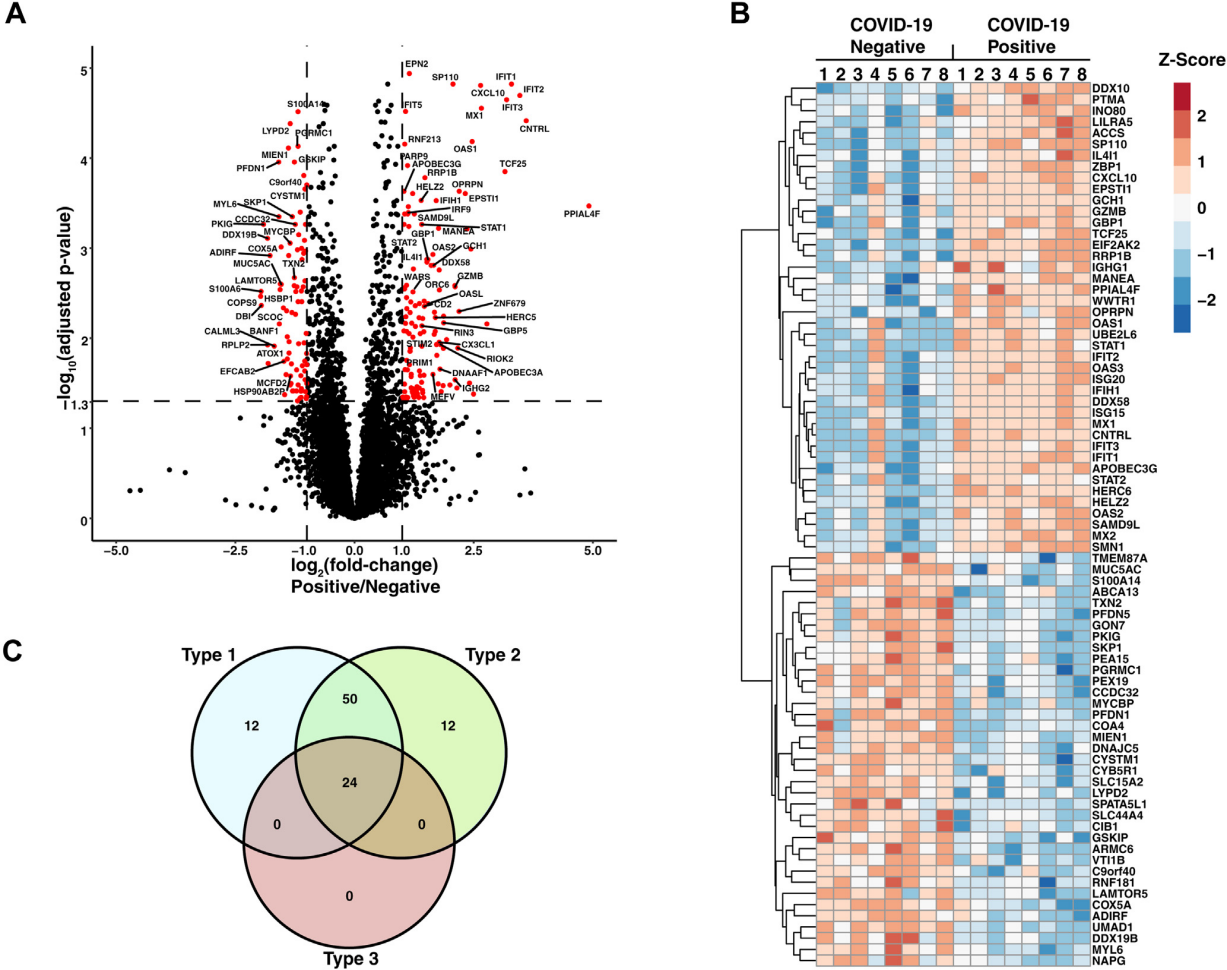
**Differential analysis of SARS-CoV-2-infected subjects and negative controls.***A*, Volcano plot displaying protein expression changes between SARS-CoV-2-infected patients (n = 8) and negative controls (n = 8). Significantly regulated proteins are colored in *red*, and rows are labeled with gene names. *B*, heatmap displaying the Z-score of select proteins with statistically significant changes in abundance between SARS-CoV-2-positive and -negative subjects. Rows are subjected to hierarchical clustering and labeled with gene names. *C*, Venn diagram indicating the number of proteins involved in type I, type II, and type III interferon (IFN) signaling. IFN signaling type was determined using the Interferome database (47). SARS-Cov-2, severe acute respiratory syndrome coronavirus 2.

To perform a multiplexed quantitative proteomic analysis, protein was extracted from NP swabs with methanol precipitation, digested with trypsin, and labeled using tandem mass tags (TMTs) (Fig. 1*B*). The labeled peptides were mixed and fractionated into 24 fractions using basic reversed-phase fractionation and subjected to LC-MS/MS analysis on an Orbitrap Fusion Lumos mass spectrometer (Thermo Fisher Scientific). After removing common contaminants and decoy sequences, we identified 7582 proteins of which 143 were upregulated and 80 were downregulated in SARS-CoV-2-infected patients (adjusted *p*-value < 0.05 and a fold-change >±2) (Fig. 2, *A* and *B*, supplemental Table S2).

### IFN-Mediated Antiviral Signaling Is Upregulated Upon SARS-CoV-2 Infection

To investigate IFN signaling in the nasopharynx of SARS-CoV-2-infected individuals, upregulated proteins were compared to a publicly available database of genes known to be increased by different types of IFNs using the web-based tool, Interferome (47). The majority of proteins that were significantly upregulated in infected subjects (70.4%) were annotated in the Interferome database as proteins upregulated upon IFN treatment in at least one dataset, confirming that these proteins are IFN responsive. These proteins include most of the classical ISGs—IFIT1/2/3, MX1/2, ISG15/20, OAS1/2, and HERC5/6 (Table 2). In addition, several molecules with different roles in IFN signaling were upregulated in infected subjects. PRRs, IFIH1 (MDA5), and DDX58 (RIG-I), which are activated by intracellular viral RNA and lead to downstream type 1 IFN signaling through mitochondrial antiviral signaling protein (MAVS), were upregulated in infected individuals. In addition, transcription factors STAT1, STAT2, and IFR9 as well as proinflammatory cytokines CXCL10 and CXCL11 were upregulated in infected individuals. Targets of type I (IFN-α/β), type II (IFN-γ), and type III (IFN-λ) IFN signaling were all upregulated in infected subjects when referenced against the Interferome database (Fig. 2*C*, Table 2). Twelve proteins were specific to type I IFN signaling, and an equal number was specific to type II IFN signaling. (Fig. 2*C*, Table 2). Most upregulated proteins, however, were known to be common mediators of multiple types of IFNs. This overlap of IFN signaling types is expected. Epithelial cells of the upper airway express type I (IFNAR) and type III (IFNLR) IFN receptors (6, 8), the latter of which are selectively expressed in epithelial tissue and play an important role in innate viral defense at barrier surfaces (8, 48). In addition, secretory and ciliated cells in airway epithelium express IFN-γ receptors (IFNGR1 and IFNGR2) and thus respond to type II IFN (IFN-γ) secreted from natural killer (NK), CD4^+^ T helper cells, and CD8^+^ cytotoxic T lymphocytes (40). It is known that the induction of target ISGs resulting from all three types of IFN signaling overlap to a great degree but differ primarily by magnitude and duration (8, 48). Therefore, IFN-mediated antiviral signaling in epithelial cells of the upper airway can result from complex interactions between all three types of IFN signaling. These signatures were detected by deep proteomic analysis of NP swabs.

**Table 2:** Upregulated interferon-stimulated genes

Gene name	Type I	Type II	Type III
*ACCS*		X	
*APCS*		X	
*APOBEC3A*	X	X	
*APOBEC3G*	X	X	
*APOL1*	X	X	
*APOL3*	X	X	
*CD5L*	X		
*CFB*	X	X	
*CMPK2*	X	X	
*CNTRL*		X	
*CX3CL1*	X	X	
*CXCL10*	X	X	
*CXCL11*	X	X	
*DCK*	X	X	
*DDX58*	X	X	X
*DDX60*	X	X	
*DDX60L*	X	X	
*DHX58*	X	X	
*DNAAF1*	X		
*EIF2AK2*	X	X	X
*EPSTI1*	X	X	X
*FGL2*	X	X	
*GBP1*	X	X	X
*GBP4*	X	X	
*GBP5*	X	X	
*GCH1*	X	X	
*GZMB*	X	X	
*H1FX*		X	
*HELZ2*	X	X	X
*HERC5*	X	X	
*HERC6*	X	X	X
*HMGB2*	X		
*HP*	X	X	
*ICAM1*	X	X	
*IDO1*	X	X	
*IFI44*	X	X	
*IFIH1*	X	X	X
*IFIT1*	X	X	X
*IFIT2*	X	X	X
*IFIT3*	X	X	X
*IFIT5*	X	X	X
*IL4I1*	X	X	
*INO80*	X		
*IRF9*	X	X	X
*ISG15*	X	X	X
*ISG20*	X	X	
*KCNAB2*	X		
*KRT13*	X		
*LCP2*	X	X	
*LILRA2*	X	X	
*LILRA5*	X	X	
*MANEA*	X		
*MEFV*	X	X	
*MKI67*	X	X	
*MT2A*	X	X	
*MX1*	X	X	X
*MX2*	X	X	
*NADK*	X	X	
*NUB1*	X	X	
*OAS1*	X	X	X
*OAS2*	X	X	
*OAS3*	X	X	X
*OASL*	X	X	X
*PARP9*	X	X	X
*PHF1*		X	
*PIK3AP1*	X		
*PIK3R3*	X	X	
*PLCD3*		X	
*PLEK*	X	X	
*PLEKHO1*		X	
*PLSCR1*	X	X	X
*PRIM1*	X	X	
*PTMA*	X		
*PTPN18*		X	
*RELB*	X	X	
*RFX5*	X	X	
*RNF213*	X	X	
*RRAGC*	X	X	
*RRP1B*		X	
*RSAD2*	X	X	
*SAMD9L*	X	X	
*SAMSN1*	X	X	
*SMCHD1*	X	X	
*SP110*	X	X	X
*SPATS2L*	X	X	
*SRSF3*	X		
*STAT1*	X	X	X
*STAT2*	X	X	X
*TF*	X		
*TGFB1*		X	
*TNFAIP8L2*		X	
*TNFSF10*	X	X	
*UBE2L6*	X	X	X
*USP18*	X	X	X
*VWA8*		X	
*WARS*	X	X	
*WIPF1*	X		
*ZBP1*	X	X	

A list of identified upregulated proteins that are known to be involved in IFN-mediated antiviral signaling (type I, II, or III) as annotated in the Interferome database (47).

**Fig. 3:**
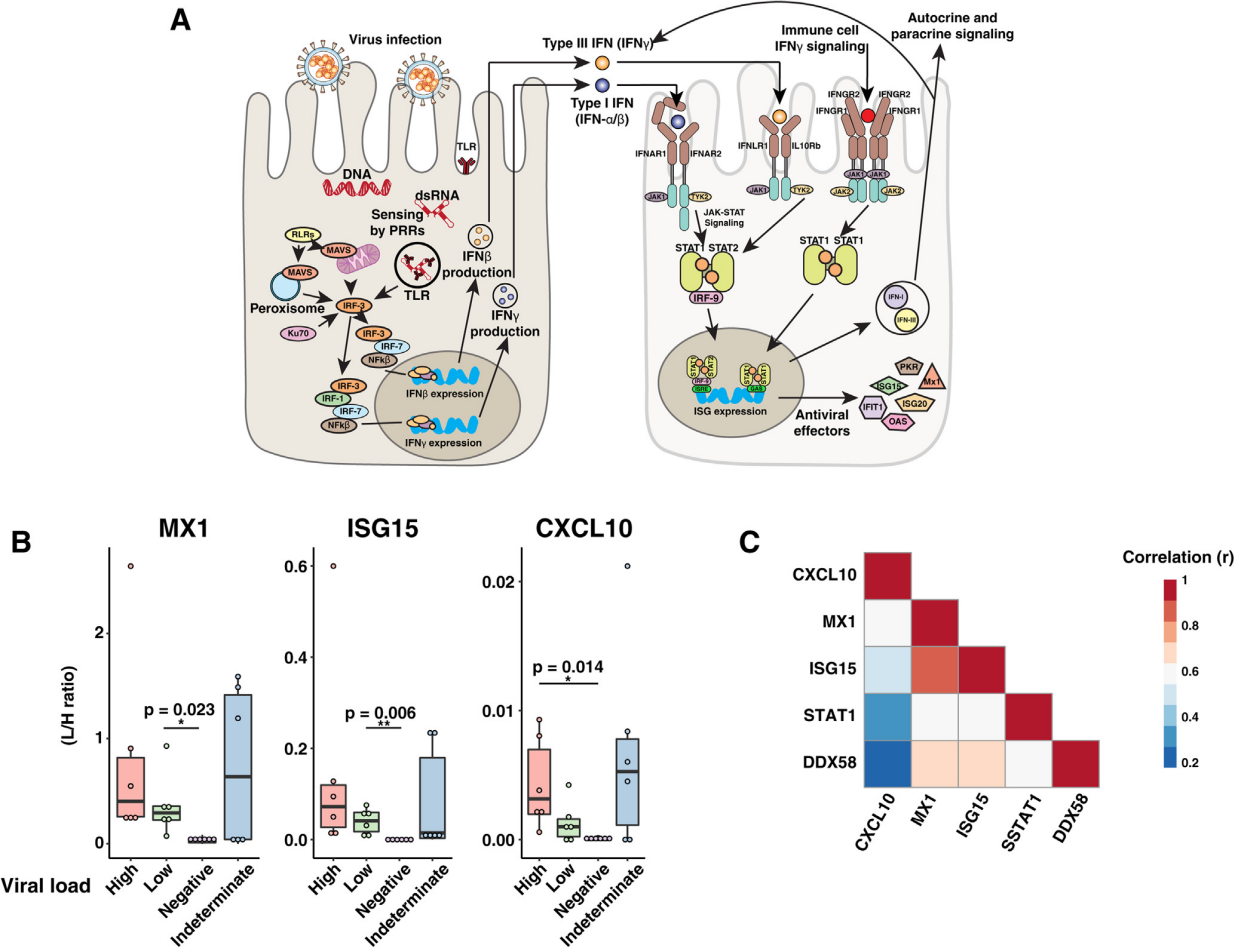
**Targeted ISG measurement.***A*, Whisker plots displaying the stabile isotope–labeled internal standard (SIL) normalized signal for each indicated protein. *B*, correlation plot indicating the correlation coefficient (r) for each indicated comparison. *C*, interferon signaling diagram. Upon detection of viral infection, epithelial cell PRRs such as RIG-I like receptors (RLRs) and toll-like receptors (TLRs) are activated. Signaling through PRRs activates IRF family transcription factors, and together with NFκB, they induce the expression of type I and type III IFNs. IFNs act in an autocrine and paracrine manner to stimulate the expression of ISGs in uninfected neighboring cells. Epithelial cells also express type II IFN receptors (IFNGR1/2) and thus respond to IFN-γ released from immune cells. IFN, interferon; ISGs, interferon-stimulated genes; PRRs, pathogen-recognition receptors.

Although some heterogeneity among subjects is noted, our findings suggest that there is a distinct IFN-mediated antiviral response in the nasopharynx of SARS-CoV-2-infected individuals and contrasts with several recent reports utilizing cell line models to study the host response to SARS-CoV-2 (38, 39, 49, 50). These studies reported that SARS-CoV-2 infection did not induce major IFN signaling but rather increased the expression of proinflammatory cytokines and chemokines. The diminished ISG expression observed upon SARS-CoV-2 infection in cell culture may indicate an impaired type I or type III IFN response. This is supported by a recent study of infected humans with varying disease severity, which found a downregulation of ISG transcripts in peripheral white blood cells of critically ill patients relative to moderately ill patients (39). The apparent discrepancy between our study and these reports may reflect the sample type used. As mentioned above, NP swabs contain a heterogeneous mixture of epithelial and immune cell subtypes as confirmed by identification of immune cell–specific (CD2, CD5, CD8, CD14, CD20, and granzyme B) and epithelial cell–specific (CDH1, ACE, CLDN3) markers in the proteomic data. The release of type I or type III IFNs from myeloid cells or IFN-γ from lymphocytes recruited to the site of infection could activate IFN receptors on epithelial cells and stimulate the expression of ISGs—this would not be observed in cell culture–based studies. These results are in agreement with a recent study that utilized single-cell RNA sequencing to analyze NP and bronchial samples to demonstrate increases in immune and epithelial cell interactions during severe SARS-CoV-2 infection (40) (Fig. 3*A*). To assess the differences of SARS-CoV-2 infection in cell culture and in humans, we compared proteins that have recently been reported to be differentially regulated upon SARS-CoV-2 infection of normal human bronchial epithelial (NHBE) cells (49) with those identified in our study. Surprisingly, only four proteins were found to be differentially regulated in both NHBE cells and NP swabs from infected humans (supplemental Fig. S1), highlighting the differences between these sample types, including the role of the microenvironment. We also compared the results of our study with a recent plasma proteomics investigation of SARS-CoV-2-infected individuals (34). This comparison found only one upregulated protein that was in common between NP swabs and plasma (supplemental Fig. S2). Our results underscore the importance of studying intact systems using infected cells harvested from the viral route of entry. These data also indicate that IFN signaling is engaged in SARS-CoV-2 subjects; however, a deficiency in type I IFN signaling could be masked by type II IFN signaling mediated by immune and epithelial cell interactions. Although IFN signaling appears to be engaged, the present study was not designed to investigate differences between various clinical outcomes. In addition, global proteomic differences in NP swabs between severe and moderate cases of COVID-19 remain to be determined.

### Targeted Mass Spectrometric Analysis of ISGs

The expression of specific ISGs has been utilized as biomarkers of disease progression, treatment response, and prognostic outcome in preclinical studies (51, 52, 53, 54, 55). Considering the wide variation in disease severity among patients infected with SARS-CoV-2, we chose to monitor two molecules involved in type I IFN signaling (RIG-I and STAT1) and three other key ISGs (MX1, ISG15, and CXCL10) using a tier 2 targeted proteomics analysis to study the biological variability of these molecules in NP swabs (Table 1). For this study, 24 independent samples with different viral loads (based on quantitative RT-PCR results) were analyzed (high viral load: n = 6, low viral load: n = 6, negative: n = 6, indeterminate: n = 6). Proteotypic target peptides were selected based on uniqueness to each protein in the human and viral (SARS-CoV-2, SARS, and MERS) proteome background. Amino acids subject to common modifications were avoided, when possible (Table 1). Stable isotope–labeled standard peptides were synthesized for all targets and spiked into samples for accurate relative comparison across groups. There was a clear increase in the monitored proteins between uninfected individuals and those who tested positive for SARS-CoV-2 (Fig. 3*B* and supplemental Fig. S3). Although a trend of increased levels in those with higher viral loads was seen, it was not statistically significant for all proteins. Interestingly, the abundance of ISGs in some of the samples which were indeterminate by RT-PCR was higher than the negative controls. Although there are no other clinical data available on the patients with indeterminate results, our findings raise the possibility that ISG abundance can detect signs of viral infection even when RT-PCR results are indeterminate. The abundance of ISGs, MX1, and ISG15 correlates well with each other and with IFN signaling molecules STAT1 and RIG-I, displaying an average Pearson correlation coefficient of 0.67 (s = 0.096) (Fig. 3*C*). In contrast, the abundance of CXCL10 did not correlate well with other ISGs, STAT1 or RIG-I (average r = 0.39, s = 0.178). To test for differences in correlation between CXCL10 and the other IFN signaling proteins, we used the R package cocor (56). This analysis found that CXCL10 abundance had a significantly lower correlation to ISG15, MX1, and RIG-I relative to correlations within each of these respective proteins (CXCL10/MX1 *versus* ISG15/MX1: *p* = 0.049, CXCL10/ISG15 *versus* ISG15/MX1: *p* = 0.016, CXCL10/RIG-I *versus* RIG-I/ISG15: *p* = 0.023, CXCL10/RIG-I *versus* RIG-I/MX1: *p* = 0.028). The CXCL10 promoter includes response elements for several transcription factors including IRF1, STAT-1, AP1, FoxA2a NF-κB, and several others (57, 58). These results may indicate that the proinflammatory cytokine CXCL10 is regulated separately from other ISGs and could potentially serve as a prognostic indicator to predict the magnitude of inflammatory response in individuals infected with SARS-CoV-2. Previous reports have shown that treatment with DS RNA and IFN-γ results in synergistic upregulation of CXCL10 in an NFκB-dependent and STAT1-independent manner (59). These results indicate that targeted analysis of IFN signaling proteins could be a valuable research tool to help elucidate mechanisms of dysregulated inflammatory response to SARS-CoV-2.

### Upregulation of proteins not directly stimulated by IFNs

We next sought to catalog proteins that are independent of the IFN signaling pathways. To accomplish this, IFN-responsive proteins in the Interferome database were filtered from the list of upregulated proteins in SARS-CoV-2-infected individuals. After filtering, a total of six proteins including WWTR1, PPIAL4F, OPRPN, LSG1, TCF25, and EPN2 were left in the list of upregulated proteins. Most of these proteins have not been extensively characterized in viral infection. WWTR1, a Hippo signaling effector protein, that acts as a transcriptional coactivator is involved in organ size control and tumor suppression by inhibiting proliferation and promoting apoptosis. Protein levels and methylation status of WWTR1 have been shown to be regulated during Zika virus infection, negatively regulating the Hippo signaling pathway (60, 61). The most highly upregulated protein in infected individuals was PPIAL4F, a peptidyl-prolyl *cis*/*trans* isomerase (PPIase), which functions to accelerate protein folding. Host PPIases have been shown to be important for efficient infection in several different viral infections, including SARS-CoV (62). In addition, the PPIases, cyclophilin A and B, have been shown to bind the capsid protein of HIV-1 (63). Opiorphin prepropeptide (OPRPN) was also upregulated in an IFN-independent manner. Opiorphin, a 5-amino acid peptide derived from the N terminus of OPRPN, has many proposed functions including inhibition of aminopeptidase N and MME (neprilysin); however, its role in viral infection is currently uncharacterized. LSG1, a GTPase involved in the biogenesis of the 60S ribosome (64), has not been studied in the context of viral infections. TCF25, a transcription factor, is also a poorly characterized component of the ribosome-associated quality control pathway that mediates K-48 linkage of ubiquitin to the nascent chain of stalled ribosomal complexes (65). Finally, EPN2 plays a role in mediating recruitment of clathrin to the cell membrane and promotes its polymerization to mediate endocytosis (66). Knockdown of EPN2 using siRNA was shown to inhibit infection of Huh7 cells by Dengue virus, indicating that it may be important in viral entry (67). The IFN-independent upregulation of these proteins makes them attractive candidates for future studies characterizing the pathogenesis of SARS-CoV-2 infection at a cellular level.

### Downregulation of Protein Synthesis

Although viruses have evolved sophisticated mechanisms to promote their proliferation, they remain completely dependent upon the host translational machinery for protein synthesis. Thus, it is not surprising that host cells respond to viral infection by inhibiting protein synthesis and disrupting mRNA transport (68, 69, 70). Consistent with these events, many proteins in SARS-CoV-2-infected individuals decreased in abundance relative to uninfected controls in our dataset. EIF2AK2, which was upregulated, is a well-known antiviral protein induced by IFNs. EIF2AK2 mediates translation inhibition of host and viral mRNAs through phosphorylation of eIF2α (69) (Fig. 1*A*). In agreement with this, we observed downregulation of several ribosomal subunits including RPLP2, MRPL12, and MRPL41. Furthermore, downregulation of host proteins has also been linked to SARS-CoV-2 nsp1, which has been shown to bind and obstruct the ribosomal mRNA entry tunnel (71), potentially exacerbating the reduction in protein synthesis.

### Regulation of the Ubiquitin–Proteasome System

The ubiquitin–proteasome system is a major intracellular protein degradation pathway present in all eukaryotes which is primarily responsible for the removal of damaged, misfolded, or unfolded proteins in the cell. The ubiquitin–proteasome system is a critical regulator of many basic cellular processes, including protein homeostasis, signaling, chromatin structure, endocytosis, development, and immunity (72). It is now well understood that certain viruses have evolved complex mechanisms to manipulate the host UPS for their own benefit (73, 74). In addition, the ubiquitin–proteasome system is required for host immune surveillance and several processes involved in viral propagation including viral genome uncoating, viral replication, and immune evasion (75, 76, 77). Interactions between the ubiquitin–proteasome system and viral proteins are typically studied by overexpressing individual viral proteins or infecting cells in culture.

Gene set enrichment analysis revealed a negative regulation of proteosomal proteins in COVID-19-positive subjects (supplemental Table S3). Further analysis revealed the downregulation of several proteasomal subunits including PSMC1, PSMD2, PSMD7, and the proteasomal ubiquitin receptor ADRM1 upon SARS-CoV-2 infection. Previous studies have implicated proteasomal proteins in viral infection. For instance, inhibition of PSMB1 was shown to positively regulate the innate immune response by increasing the production of IFN-β and proinflammatory cytokine TNF-α (78). Wholesale downregulation of proteasomal degradation, however, is a less commonly observed host response to viral infection. Some studies have investigated the use of proteasome inhibitors as antiviral agents with promising results (79, 80, 81, 82), suggesting that proteasomal downregulation may be a host viral defense measure. The effect of proteasomal downregulation with SARS-CoV-2 infection remains to be determined and further studies are required to determine if this is a host- or viral-mediated adaptation.

Over the course of evolution, viruses have adapted to use the ubiquitylation process to favor conditions that promote their survival and replication. In fact, several viruses encode E3 ligases to target specific host proteins for degradation affecting processes including antigen presentation IFN-mediated antiviral defense (83, 84, 85, 86). We identified a number of differentially regulated E3 ubiquitin-protein ligases in our data. ISG15, a ubiquitin-like protein that mediates antiviral activity *via* ISGylation of target proteins during viral infection, and its E3 ligases, HERC5 and UBE2L6, are upregulated by SARS-CoV-2 infection (87). We also observed the regulation of several RING domain E3 ligases. RING domain E3 ligases are involved in several aspects of immune function including regulation of RIG-I signaling and cell surface expression of MHC molecules. RNF213 was upregulated 2-fold, and RNF181 and RNF126 were downregulated 2-fold in these data. RNF213 is involved in angiogenesis (88, 89, 90, 91) and could be related to the observed vascular abnormalities in COVID-19 patients (92, 93). The role of RNF181 and RNF126, which are downregulated, is not understood although our data suggest that they may mediate SARS-CoV-2-induced signaling.

### Phosphoproteomics Analysis

**Fig. 4:**
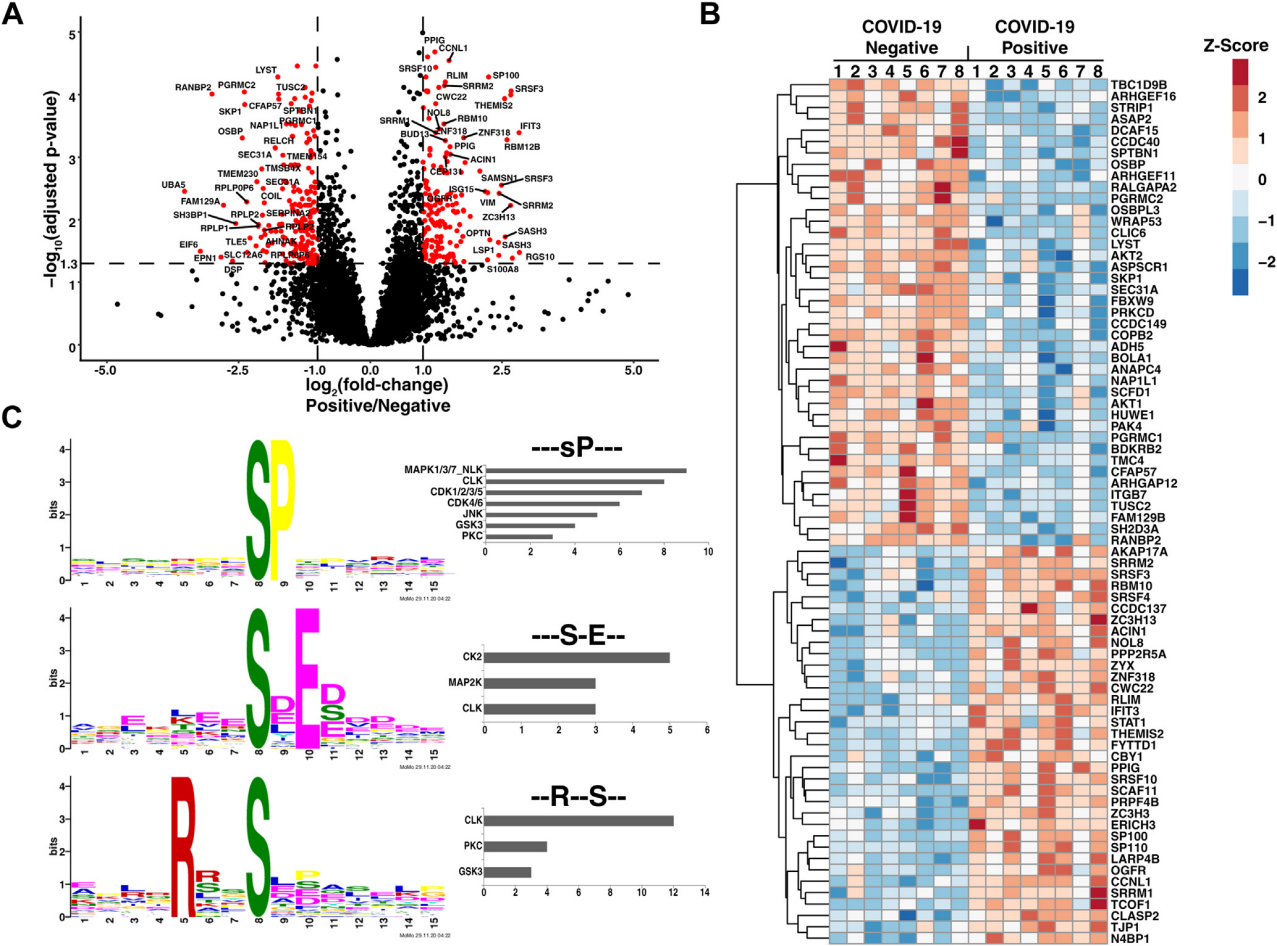
**Quantitative phosphoproteomics.***A*, Volcano plot illustrating changes in phosphosite abundance between COVID-19-positive (n = 8) and negative controls (n = 8). Phosphosites with a positive fold change are upregulated in COVID-19-positive subjects, and significantly regulated sites are colored in *red*. *B*, heat map depicting relative abundance changes in abundance for select significantly regulated phosphosites. *C*, sequence logos representing enriched phosphorylation motifs identified in the downregulated phosphosites using the motif-x algorithm. Predicted upstream kinases for phosphosites that contain the enriched motifs are plotted in a bar chart (*right*). COVID-19, coronavirus disease 2019.

To study the impact of SARS-CoV-2 infection on host signaling, we also undertook a phosphoproteomics analysis of NP swab samples. TMT-labeled tryptic peptides were enriched with immobilized metal affinity chromatography (IMAC) and analyzed by LC-MS/MS. This analysis identified >8500 phosphorylation sites, of which 194 were upregulated and 213 were downregulated (>2-fold) (Fig. 4, *A* and *B*, supplemental Table S4). These changes were mainly regulated at the phosphorylation level (i.e., the protein level was unchanged) (supplemental Table S5). Regulated phosphorylation sites were identified in twenty different kinases with an FDR <0.1% (Table 3), five of which were upregulated and 15 downregulated in infected subjects relative to uninfected controls. Similar to the global proteomics data, a comparison of regulated phosphosites between NHBE cells infected with SARS-CoV-2 (49) and the data from our study illustrates markedly different sets of regulated molecules between the two samples types (supplemental Fig. S1), highlighting the importance of studying host response using both cell line models and cells harvested from infected subjects.

**Table 3:** A list of significantly regulated kinases

Gene symbol	Protein	Adjusted *p*-value	Fold change (log_2_)	Amino acid	Position	Localization probability
*PRPF4B*	Serine/threonine-protein kinase PRP4 homolog	0.022	1.7	S	61	0.829
*PRPF4B*	Serine/threonine-protein kinase PRP4 homolog	0.005	1.4	S	349	1.000
*PRPF4B*	Serine/threonine-protein kinase PRP4 homolog	0.018	1.3	S	341	1.000
*SNRK*	SNF-related serine/threonine-protein kinase	0.008	1.2	S	584	0.934
*SCYL2*	SCY1-like protein 2	0.085	1.1	S	3	1.000
*FGR*	Tyrosine-protein kinase Fgr	0.045	1.1	S	261	0.955
*STK10*	Serine/threonine-protein kinase 10	0.055	1.0	S	454	0.867
*PRPF4B*	Serine/threonine-protein kinase PRP4 homolog	0.000	1.0	S	257	1.000
*PRPF4B*	Serine/threonine-protein kinase PRP4 homolog	0.017	1.0	S	387	0.999
*ERBB2*	Receptor tyrosine-protein kinase erbB-2	0.001	-1.1	S	1054	1.000
*STK25*	Serine/threonine-protein kinase 25	0.050	-1.1	T	174	0.831
*STK39*	STE20/SPS1-related proline-alanine-rich protein kinase	0.001	-1.1	S	385	1.000
*AKT1*	RAC-alpha serine/threonine-protein kinase	0.009	-1.1	S	126	0.756
*MAST2*	Microtubule-associated serine/threonine-protein kinase 2	0.042	-1.3	S	900	1.000
*MAP3K5*	Mitogen-activated protein kinase kinase kinase 5	0.012	-1.3	S	1033	0.899
*AKT2*	RAC-beta serine/threonine-protein kinase	0.000	-1.3	T	451	0.990
*STK25*	Serine/threonine-protein kinase 25	0.006	-1.4	T	168	0.923
*PAK4*	Serine/threonine-protein kinase PAK 4	0.000	-1.5	S	474	0.998
*PRKCD*	Protein kinase C delta type	0.001	-1.5	S	664	1.000
*FRK*	Tyrosine-protein kinase FRK	0.050	-1.5	Y	497	0.942
*SLK*	STE20-like serine/threonine-protein kinase	0.055	-1.6	T	183	0.855
*ATM*	Serine-protein kinase ATM	0.053	-1.7	S	2209	1.000
*MAP3K6*	Mitogen-activated protein kinase kinase kinase 6	0.055	-1.9	S	1149	0.942
*TRIM24*	Transcription intermediary factor 1-alpha	0.048	-2.0	S	811	0.784
*PKN2*	Serine/threonine-protein kinase N2	0.087	-5.6	S	306	0.796

Kinases regulated with a log2 fold change greater than ±1 and an adjusted *p*-value < 0.1 are presented.

Phosphosites on several proteins involved IFN signaling, RNA processing, and protein synthesis were differentially regulated in infected individuals. Increased phosphorylation of serine 478 on IFIT3, a well-characterized ISG with antiviral effects, was observed in infected subjects. In addition, increased phosphorylation sites on two RNA-binding proteins, RBM12B and RBM10, were also observed in infected individuals. Furthermore, phosphosites on several proteins involved in protein translation including EIF6, EIF4EBP1, and EIF4G3 were downregulated in infected subjects. The observed increase in RNA binding and decrease in protein synthesis likely reflect an IFN-mediated antiviral cellular state that is meant to decrease the translation of host and viral proteins. Currently, there are no comprehensive resources to evaluate phosphorylation events in the context of viral pathogenesis. To investigate the role of some interesting molecules that displayed regulated phosphorylation sites, we performed a manual literature search. CEP131, a component of pericentriolar satellites that regulates cilia and flagellum formation, displayed increased levels of phosphorylation on S78 in infected subjects. CEP131 is phosphorylated on Ser 78 by PLK4 to maintain centriolar satellite integrity (94); however, its role in viral infection is unknown. In addition, phosphorylation of Ser 1237 in CFAP57 was decreased in COVID-19-positive subjects relative to negative controls. CFAP57 is a conserved component of motile cilia and flagella (95, 96) that has been shown to form a complex connecting components critical for motility (97). Cilia and flagella are essential structures for the sense of smell, and the regulated phosphorylation of CEP131 and CFAP57 could perhaps be related to the loss of smell (anosmia) associated with SARS-CoV-2 infection.

### Downregulation of AKT and PKC Phosphorylation

AKT signaling regulates many critical cellular processes and has been targeted by many viruses for their benefit with widely varying mechanisms (98). For instance, influenza A has been shown to activate AKT signaling to inhibit apoptosis (99, 100, 101, 102). In contrast, measles virus can downregulate AKT signaling (103), highlighting the diverse mechanisms that viruses use to interact with the host system for their benefit. In this dataset, infected subjects displayed decreased levels of Ser 126 phosphorylation in AKT1 and Thr 451 phosphorylation in AKT2. Isoform-specific phosphorylation of AKT1 and AKT2 has been previously described (104), and phosphorylation of Ser 451 on AKT2 is known to positively regulate AKT signaling (104, 105). The observed downregulation of AKT signaling in infected subjects is also in agreement with a previous report utilizing SARS-CoV-2-infected Vero E6 cells (35). The precise mechanism of AKT downregulation during SARS-CoV-2 infection remains to be determined, although the importance of this signaling pathway in regulating critical cellular processes suggests that AKT signaling downregulation by SARS-CoV-2 may play an important role in the course of infection.

PKC signaling has a wide range of effects on cellular signaling and has been shown to be involved in viral entry (106). PKC-δ serine 664 phosphorylation was downregulated in infected subjects. The role that PKCs play in viral replication has been controversial although some reports have shown that pharmacological inhibition of PKC signaling reduces replication of West Nile virus (107). Downregulation of PKC-δ signaling in infected subjects could potentially be a host adaptation to limit viral entry; however, further experiments will be required to elucidate the biological relevance of this phosphorylation event in the context of SARS-COV-2 infection.

### Opioid Growth Factor Receptor Signaling

Phosphorylation of S55 on the OGFR was upregulated 2.2-fold in infected subjects. Phosphorylation of this serine residue has been previously observed although its regulatory role remains unclear (108, 109, 110). When the OGFR binds to its ligand, it negatively regulates cell growth by inhibiting G1-S transition. This action is mediated through OGFR-dependent upregulation of CDK inhibitors p12 and p16, which inhibits CDK4-mediated phosphorylation of retinoblastoma protein (111, 112). Although neither p12 nor p16 was significantly upregulated in infected subjects, we observed that one of three enriched phosphorylation motifs in the downregulated phosphosites was predicted to be phosphorylated by CDKs 1 to 4 (Fig. 4*C*), thereby providing a possible link between OGFR signaling and cell growth arrest upon SARS-CoV-2 infection.

OPRPN, whose protein level was increased, has been shown to have many effects although it is most known for its inhibitory effects on the endopeptidases neprilysin and aminopeptidase-N. Neprilysin, a type II transmembrane glycoprotein that inactivates several peptide hormones including glucagon, met-enkephalin, bradykinin, substance P, angiotensin 1, and angiotensin 2, was downregulated in SARS-CoV-2-positive subjects. Opiates and endogenous opioid peptides are known to have immunomodulatory effects and previous reports have linked OGFR signaling to immunosuppression of T-lymphocytes (113). The observed upregulation of OPRN together with the increased phosphorylation of the OGFR on S55 and decreased levels of neprilysin may indicate that the OGFR signaling axis plays a role in response to SARS-CoV-2 infection and warrants further investigation.

## Conclusions

In the present study, we used global proteomics and phosphoproteomics to characterize the host response to SARS-CoV-2 directly at the point of infection using NP swabs. Infected subjects displayed upregulation of 98 proteins known to be stimulated by IFNs, which included classical ISGs (MX1, MX2, ISG15, IFIT1, OAS1 and IDO), PRRs (MDA5, DDX58), transcription factors (IRF9, STAT1, STAT2), and proinflammatory cytokines (CXCL10 and CXCL11). In contrast to previously published studies that mimicked SARS-CoV-2 infection using cultured cell lines, IFN-mediated antiviral responses appear to be robust in the nasopharynx of infected subjects. The discrepancy between previous cell line studies and the clear evidence of type I and type II IFN-mediated effects presented here indicates that epithelial and immune cell interactions in the microenvironment is required to mediate the entire spectrum of host responses to SARS-CoV-2 infection. Targeted analysis of IFN signaling molecules STAT1 and RIG-I along with the key ISGs, MX1, ISG15, and the proinflammatory cytokine CXCL10 demonstrate that levels of these antiviral proteins generally correlate with the viral loads. Although CXCL10, also known as IFN γ-induced protein 10 kDa (IP-10), was upregulated, its correlation with the levels of other ISGs was not high and needs to be investigated further. Other features of SARS-CoV-2 infection apparent from this study include the upregulation of multiple proteins that are not IFN responsive and have not been well-characterized during viral infection. In addition, downregulation of several proteasomal proteins and the regulation of several E3 ubiquitin ligases were observed in infected subjects. Phosphoproteomic profiling indicates that several signaling pathways including AKT, PKC, and OGFR are affected and may play a role in SARS-CoV-2 pathology. The present study was not designed to detect differences between patients with clinical outcomes, and further studies focusing on these features in individuals with varying outcomes or infection from different viruses may shed additional light on the pathogenesis of COVID-19.

## Data availability

The mass spectrometry proteomics data have been deposited to the ProteomeXchange Consortium *via* the PRIDE (114) partner repository with the dataset identifier PXD022889.

## Supplemental data

This article contains supplemental data (34, 49).

## Conflict of interest

The authors declare no competing interests.

## Supplemental data

Supplemental Fig. S1 **Comparison of regulated proteins in NHBE cells and NP swabs upon SARS-CoV-2 infection.***A*, Venn diagram comparing differentially regulated proteins and phosphosites (*B*) observed upon SARS-CoV-2 infection of NHBE cells (49) with those differentially regulated in NP swabs from COVID-19-positive subjects in the present study. COVID-19, coronavirus disease 2019; NHBE, normal human bronchial epithelial; NP, nasopharyngeal; SARS-Cov-2, severe acute respiratory syndrome coronavirus 2.

Supplemental Fig. S2 **Comparison of regulated proteins in plasma and NP swabs upon SARS-CoV-2 infection**. *A* and *B*, Venn diagram comparing upregulated (*A*) or downregulated (*B*) proteins identified in plasma by Overmyer *et al*. (34) and NP swabs from the present study in SARS-CoV-2-infected individuals. NP, nasopharyngeal; SARS-Cov-2, severe acute respiratory syndrome coronavirus 2.

Supplemental Fig. S3 **Targeted analysis of interferon signaling molecules STAT1 and RIG-I.** Whisker plots displaying the stable isotope–labeled internal standard (SIL) normalized signal for each indicated protein. Significant differences in comparisons between high viral load, low viral load, and negative controls are indicated with an *asterisk*.

Supplemental Table S1 Summary of subject characteristics. Cycle threshold (CT Value), age and gender of the COVID-19 positive subjects.

Supplemental Table S2 List of identified proteins. All identified proteins are listed with their corresponding quantitation results and identification information.

Supplemental Table S3 Gene Set Enrichment Analysis (GSEA). Gene sets that are positively enriched (Sheet 1) or negatively enriched (Sheet 2) in the COVID-19 positive subjects (FDR adjusted *p*-value < 0.05).

Supplemental Table S4 List of identified phosphosites. All identified phosphosites are listed with their corresponding quantitation results and identification information.

Supplemental Table S5 Combined table of protein level and phosphorylation level changes. All regulated phosphosites with an adjusted p-value (FDR) of <0.1 are displayed with their corresponding protein level changes. Phosphosites without a corresponding protein level change are indicated as NA in the protein p-value, FDR and log_2_(fold-change) columns. Phosphosites which are also regulated at the protein level are highlighted in red.
