# Supplementary figures and images for: Proteomic Signature of Host Response to SARS-CoV-2 Infection in the Nasopharynx
Source: Mol Cell Proteomics. 2021 Aug 14;20:100134. doi: 10.1016/j.mcpro.2021.100134 (PMC8363427; doi:10.1016/j.mcpro.2021.100134)

**A**

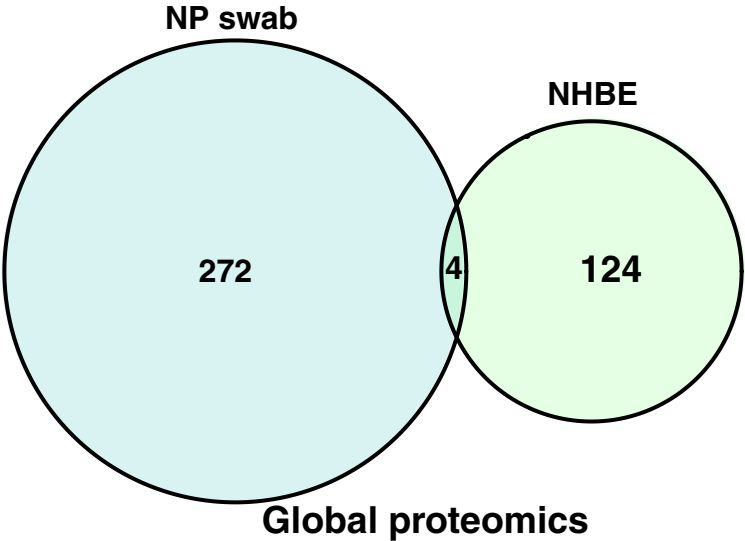

**B**

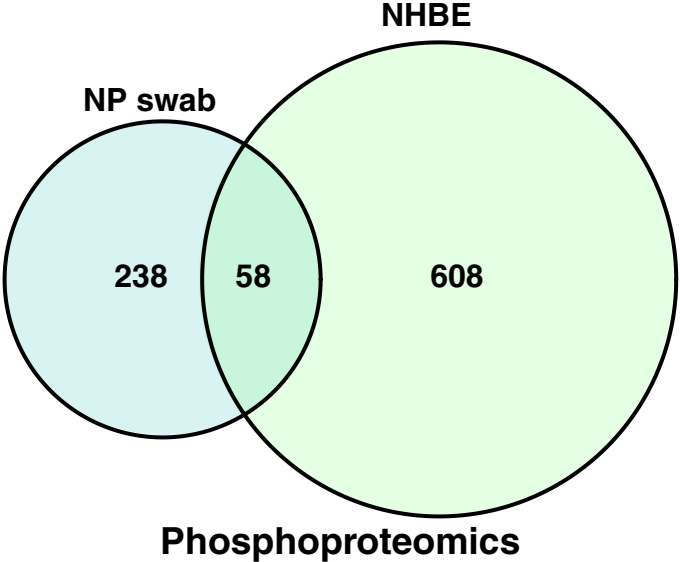

Supplement: Supplemental Fig. S1 — Comparison of regulated proteins in NHBE cells and NP swabs upon SARS-CoV-2 infection.A, Venn diagram comparing differentially regulated proteins and phosphosites (B) observed upon SARS-CoV-2 infection of NHBE cells (49) with those differentially regulated in NP swabs from COVID-19-positive subjects in the present study. COVID-19, coronavirus disease 2019; NHBE, normal human bronchial epithelial; NP, nasopharyngeal; SARS-Cov-2, severe acute respiratory syndrome coronavirus 2. [file mmc1.pdf]

A

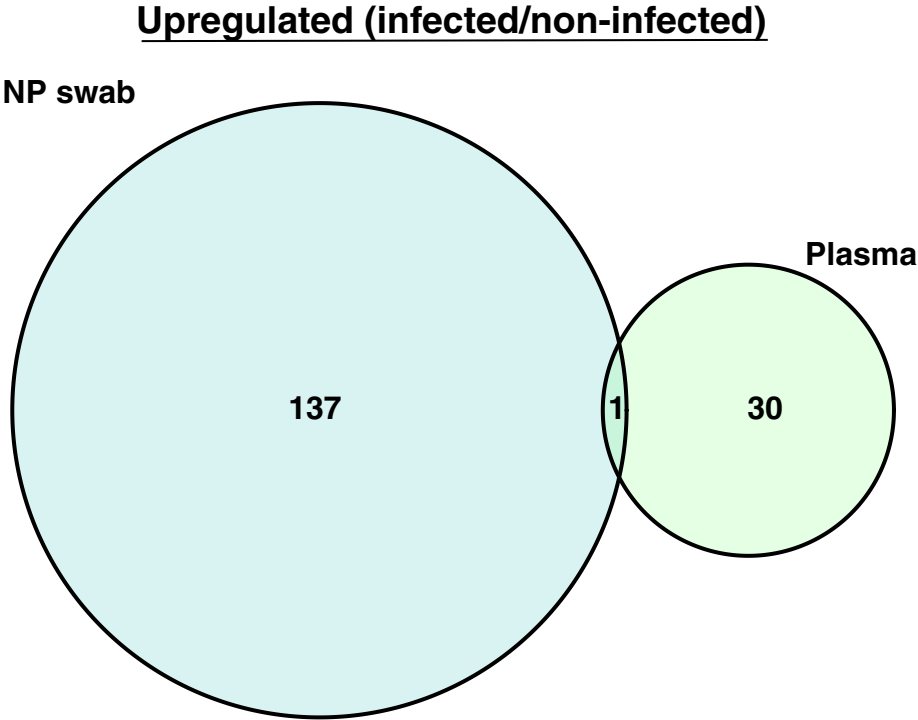

B

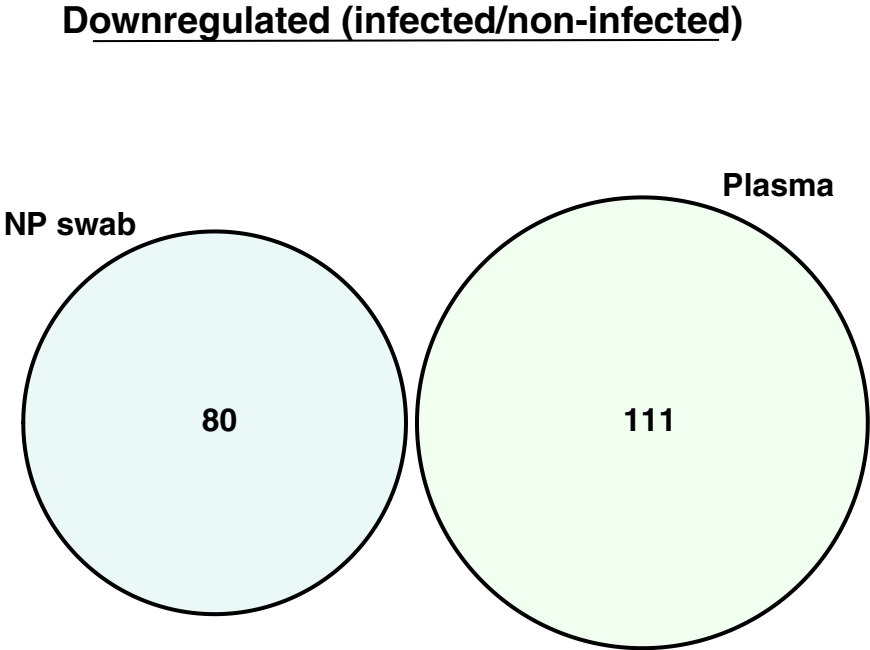

Supplement: Supplemental Fig. S2 — Comparison of regulated proteins in plasma and NP swabs upon SARS-CoV-2 infection. A and B, Venn diagram comparing upregulated (A) or downregulated (B) proteins identified in plasma by Overmyer et al. (34) and NP swabs from the present study in SARS-CoV-2-infected individuals. NP, nasopharyngeal; SARS-Cov-2, severe acute respiratory syndrome coronavirus 2. [file mmc2.pdf]

Figure S3

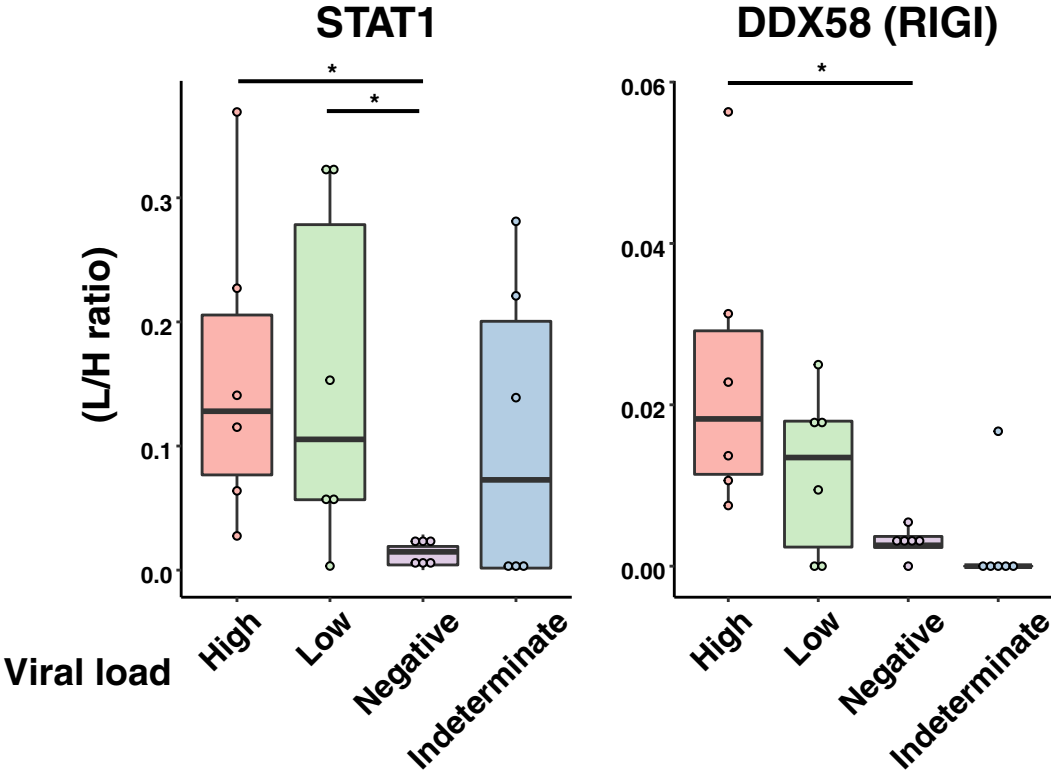

Supplement: Supplemental Fig. S3 — Targeted analysis of interferon signaling molecules STAT1 and RIG-I. Whisker plots displaying the stable isotope–labeled internal standard (SIL) normalized signal for each indicated protein. Significant differences in comparisons between high viral load, low viral load, and negative controls are indicated with an asterisk. [file mmc3.pdf]
